# Hydrogel-Based Controlled Delivery Systems for Articular Cartilage Repair

**DOI:** 10.1155/2016/1215263

**Published:** 2016-08-23

**Authors:** Ana Rey-Rico, Henning Madry, Magali Cucchiarini

**Affiliations:** ^1^Center of Experimental Orthopaedics, Saarland University Medical Center, Homburg, 66421 Saar, Germany; ^2^Department of Orthopaedic Surgery, Saarland University Medical Center, Homburg, 66421 Saar, Germany

## Abstract

Delivery of bioactive factors is a very valuable strategy for articular cartilage repair. Nevertheless, the direct supply of such biomolecules is limited by several factors including rapid degradation, the need for supraphysiological doses, the occurrence of immune and inflammatory responses, and the possibility of dissemination to nontarget sites that may impair their therapeutic action and raise undesired effects. The use of controlled delivery systems has the potential of overcoming these hurdles by promoting the temporal and spatial presentation of such factors in a defined target. Hydrogels are promising materials to develop delivery systems for cartilage repair as they can be easily loaded with bioactive molecules controlling their release only where required. This review exposes the most recent technologies on the design of hydrogels as controlled delivery platforms of bioactive molecules for cartilage repair.

## 1. Introduction

Articular cartilage is an avascular tissue that lines the weight-bearing surface of joints, formed of organized populations of chondrocytes surrounded by their extracellular matrix (ECM) (proteoglycans, type II collagen) and regulated by a metabolic balance that involves diverse signaling molecules, growth factors, and cytokines [1]. Due to the lack of access to blood supply, the cartilage has a limited ability to self-heal and full repair of cartilage defects is thus a major clinical challenge that may progress to osteoarthritis [2–4], a critical disorder affecting a large number of patients. While various options are available to repair injured cartilage (marrow-stimulating techniques, transplantation of tissue or cells as autologous chondrocytes or mesenchymal stem cells (MSCs), and replacement surgery) [5–8], still none of them are capable of reproducing the natural functions of the native, hyaline cartilage, rather leading to the formation of a poorly mechanically functional fibrocartilaginous surface (type I collagen). In this sense, large efforts are ongoing to improve these procedures and considerable progress has been made in the last few years by identifying novel methods and factors that may stimulate the reparative activities in sites of cartilage injury. Nevertheless, regenerating the desirable phenotypic response from host and/or codelivered progenitor cells remains a major issue in orthopaedics.

Tissue engineering and regenerative medicine approaches based on the design of biomaterials scaffolds reflecting the properties of the native cartilage may provide potent alternatives to restore a healthy and fully functional articular cartilage. Thus far, advances in biomedical devices for controlled drug delivery platforms support a new generation of products to treat such disorders where a temporal control over the pharmacokinetic profiles is required [9]. Hence, the use of biomaterials as controlled delivery systems has shown to be a powerful strategy to improve the temporal and spatial presentation of therapeutic agents in a defined target protecting the cargo from physiological degradation [10, 11]. Most advanced tissue engineering approaches for cartilage repair involve a combination of scaffolds with optimal properties, cells relevant to the tissue (chondrocytes, MSCs), and biological cues (growth factors/cytokines or therapeutic gene transfer vectors). Hydrogel-based biomaterials are promising compounds for cartilage repair not only because of their high water content like in the native cartilage but also because they can be typically fabricated under mild conditions, enabling the encapsulation of labile biomolecules such as growth factors [12].

The goal of the present work is to provide an overview of the most recent advances in the preparation of polymeric hydrogel networks as controlled delivery systems of the most relevant bioactive molecules involved in articular cartilage repair.

## 2. Hydrogels in Cartilage Tissue Engineering

### 2.1. Features of Hydrogels

Hydrogels are polymeric networks consisting of cross-linked hydrophilic polymers able to swell and retain a significant fraction of water within their structure but will not dissolve in water [13]. The ability of hydrogels to absorb water arises from hydrophilic functional groups attached to the polymeric backbone while their resistance to dissolution is a result of the cross-links between network chains. In general, hydrogels are characterized by good biocompatibility, high permeability for oxygen and nutrients, production under mild conditions, and ease of cell encapsulation resulting in a homogeneous distribution [12]. Hydrogels are particularly attractive biomaterials for cartilage repair as they reflect the diverse properties of native cartilage and can be provided in a minimally invasive way to fill defects of any size [14].

Hydrogels can be classified into physical and chemical systems based on their cross-linking mechanism [15, 16]. While physical cross-links involve the entanglement of the chains by hydrogen bonding, hydrophobic interaction, and crystallite formation, chemical (or covalent) cross-links are permanent junctions formed by covalent bonds [16]. Regarding their nature, hydrogels can be categorized as natural, synthetic, or natural/synthetic hybrid biomaterials as a function of the origin from the polymers used for their fabrication [15]. Natural hydrogels have high biocompatibility and inherent biodegradability and are commonly used in cartilage tissue engineering due to intrinsic prochondrogenic properties and involvement in native cellular processes [16, 17]. The most widely exploited natural polymers for cartilage tissue engineering include alginate [18–22], hyaluronic acid (HA) [23, 24], and fibrin [25–30]. Synthetic hydrogels have more reproducible physical and chemical properties than natural polymers but they lack such cell bioactivity properties [16]. Synthetic hydrogels can be designed to be “smart” or stimuli-sensitive polymers, having the ability to swell or deswell in response to small changes in the environment such as temperature, pH, or ionic strength [31, 32]. Some of the most extensively used synthetic polymers for cartilage repair include polyester copolymers from poly(lactic acid) as poly(lactide-co-glycolide) (PLGA) [33, 34], self-assembling peptides [35–37], nonbiodegradable polymers as polyethylene glycol (PEG) [38–42], thermoreversible polymers such as poly(N-isopropylacrylamide) (pNIPAAm) [43], and polyethylene oxide (PEO) and polypropylene oxide- (PPO-) based copolymers (poloxamers or Pluronic® and poloxamines or Tetronic®) [22, 44].

### 2.2. Hydrogels as Controlled Delivery Systems for Cartilage Repair

The use of hydrogels as controlled delivery systems of bioactive molecules in strategies of cartilage repair aims at reproducing the complex microenvironment that naturally occurs in articular cartilage in an artificial setting [45]. Control over delivery of the therapeutic factors can be achieved by tuning physical properties from the hydrogels such as pore size and degradation kinetics [16]. Appropriate pore size and density are key parameters to modulate the release of the bioactive molecules and to ensure the accommodation of viable cells for cartilage tissue engineering approaches, involving the immobilization of cells within the scaffold. Biodegradability of the hydrogels is also another essential feature as it should be orchestrated by the new tissue formation rate.

Controlled delivery systems have been conceived to modify certain parameters of the biomolecule to be delivered including its release profile, ability to cross biological barriers, biodistribution, clearance, and stability (metabolism) [46]. Hence, the therapeutic efficiency of the bioactive molecule will strongly depend on the success rate of the active substances to reach the target site. While the easiest way to achieve cartilage repair is probably based on the direct intra-articular injection of a therapeutic composition, the frequency and (supraphysiological) levels of the therapeutic dose required, plus the possible diffusion of the treatment to nontarget sites and potential neutralization by host inflammatory or immune responses, often hinder the overall efficacy of the approach [11, 47].

Another key parameter when designing a hydrogel-based controlled delivery system for cartilage repair is the mechanism of loading of the bioactive substance into the hydrogel network, having a strong influence on its release profile and subsequently on its therapeutic action. Incorporation of bioactive substances within hydrogel networks can be performed by physical encapsulation, physical or chemical immobilization of the biomolecules to the polymeric network, and electrostatic interaction [45]. Physical encapsulation is a mild approach to load a molecule into the polymeric network preventing its denaturalization [48]. By this method, the release profile of the bioactive factor is mainly controlled by diffusion of the molecule through the pores of the hydrogel network but often results in a short-term release. Chemical or physical immobilization of biomolecules in a hydrogel network involves a release profile controlled by polymer degradation, linker, or by dissociation from the gel matrix. Even though a long-term release profile of the therapeutic molecule is usually achieved with this method, the harsh conditions involved here to immobilize the molecule within the hydrogel network can produce its denaturalization. Thus far, strong chemical bonds can lead to an incomplete release of the molecule from the hydrogel [45, 49].

**Figure 1:**
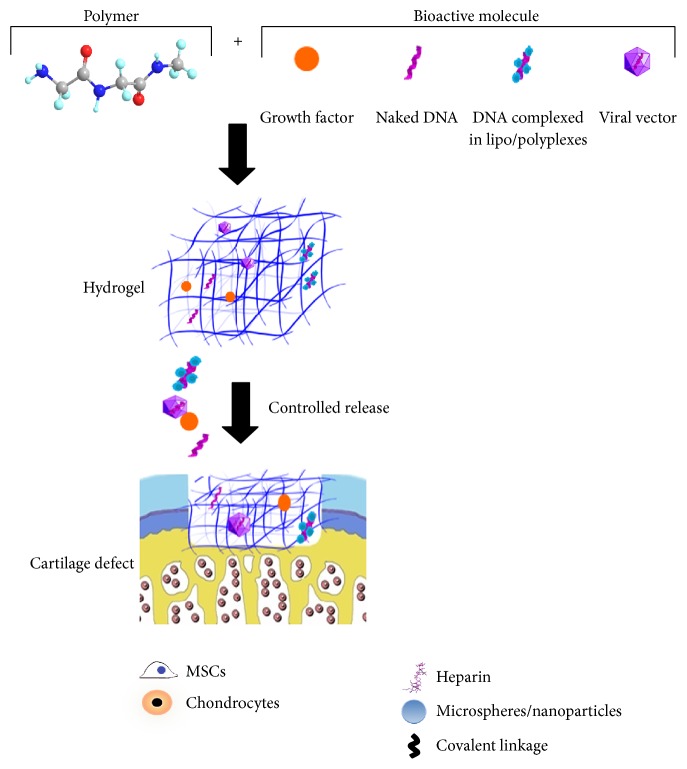
Overview of the main strategies used to design hydrogel-based delivery systems for cartilage repair. Chondroreparative factors (growth factors, nonviral gene transfer vectors including naked DNA or DNA complexed in lipo/polyplexes, and viral gene transfer vectors) may be encapsulated or immobilized into hydrogel networks by exploiting different properties from the biomolecule itself, as the affinity for heparin from growth factors. Delivery systems can be endowed with relevant populations for cartilage repair such as MSCs and chondrocytes or directly implanted as cell-free constructs into the cartilage defects providing a sustained release profile of the therapeutic factor.

An overview of the most relevant strategies used for the fabrication of hydrogels for controlled release of bioactive substances for cartilage repair is presented in the following section (Figure 1).

## 3. Controlled Delivery of Bioactive Factors from Hydrogels for Cartilage Repair

Hydrogel-based delivery systems have been used for the controlled release of bioactive factors, having a pivotal role in cartilage regeneration processes such as growth factors [12] and gene transfer vectors [47].

### 3.1. Growth Factors

Growth factors are polypeptides involved in the cellular communication system, capable of transmitting signals that modulate cellular activity by either stimulating or inhibiting cellular proliferation, migration, differentiation, and/or gene expression [50, 51]. Growth factors have a pleiotropic nature; that is, the same growth factor may act on different cell types inducing similar or different effects. Growth factors are usually produced by the cells as inactive or partially active precursors. They can be activated upon proteolytic cleavage or by binding to the ECM [50, 51]. Hence, growth factors must be delivered into the target place to be active as their rapid inactivation in physiological conditions (half-life in range of minutes) compromises their capacity to reach the cellular ECM. As a matter of fact, single doses of growth factors are often ineffective and large supraphysiological doses with the subsequent risks of severe adverse effects are required to initiate the healing of cartilage defects [12, 52, 53]. Thus, the use of hydrogels that provide a temporal or spatiotemporal control of the growth factor may be a valuable strategy to circumvent such limitations. Among the most widely used growth factors involved in cartilage repair are members of the transforming growth factor (TGF-*β*) superfamily [12] or bone morphogenetic proteins (BMP) [54], basic fibroblast growth factor (FGF-2) [55], and insulin-like growth factor I (IGF-I) [56].

Controlled delivery of growth factors from hydrogels may occur over an extended time, reducing the need for additional applications of the protein. Moreover, strictly localized release of growth factors may confine their activity to a distinct location in the proximity of the defect site reducing potential side effects [57]. Delivery of growth factors from hydrogels can be modulated by tuning different properties from the biomaterial as cross-linking density changes the free space for diffusion of the bioactive molecule [58]. Other strategies include the modification of the interaction of the growth factor with the hydrogel network as charge interactions [58, 59]. Controlled release of growth factors for cartilage repair focuses on the targeting of relevant cell populations involved in cartilage regenerative processes, specifically chondrocytes and MSCs. A compilation of the main biomaterials used to produce hydrogels in cartilage repair as well of the strategies involved to incorporate the growth factors and their release profile from the polymeric network is given in Table 1.

Natural polymers such as alginate [18, 19], fibrin [25–28, 30], HA [23, 24], and chitosan [60] have been widely used to produce hydrogel-based delivery systems of growth factors due to the optimal properties from these biomaterials mimicking the natural ECM from cartilage. Encapsulation into fibrin hydrogels is a valuable strategy to develop controlled delivery systems for cartilage repair as diffusion from the biomolecules can be modulated by modifying some parameters from the hydrogel network as fibrinogen component or thrombin concentration [26] and has been used for controlled release of IGF-I [25, 27] and TGF-*β*1 [26] both* in vitro* to target MSCs [26] or in an osteochondral defects model* in vivo* [25, 27], showing effectiveness in promoting cartilage regenerative processes. An advantageous strategy for controlling the delivery of growth factors involves the heparinization of hydrogel to immobilize the growth factor by binding to their heparin-binding domains that enable linkage with their receptors [61]. This strategy has been used for immobilization of TGF-*β*1 [28] or BMP-2 [23] in fibrin [28] and HA [23] hydrogels.

Synthetic polymers such as PEG [33, 38–40, 42], self-assembling peptides [35, 36], pNIPAAm [43], and poly(vinyl alcohol) (PVA) [34] have been used to prepare hydrogels as delivery systems of growth factors in cartilage tissue engineering approaches. PEG is the most investigated polymer for hydrogel production due to its good solubility in water and in organic solvents and lack of toxicity [33, 38–40, 42]. Incorporation of growth factor loaded in microspheres into interconnected PEG-based hydrogels is an attractive strategy to simultaneously achieve a sustained release of the protein and an adequate microenvironment for chondrogenesis [62]. Park et al. [39] embedded bovine chondrocytes into oligo(poly(ethylene glycol) fumarate) (OPF) composite hydrogels coencapsulating gelatin microparticles loaded with TGF-*β*1. Controlled release of TGF-*β*1 from the constructs increased cellularity with maintenance of the cell phenotype [39].

Most advanced strategies focused on the dual release of growth factors to achieve a synergistic effect on the enhancement of chondrogenic differentiation and on the maintenance of their phenotype. Holland et al. [40] investigated the local delivery of TGF-*β*1 and IGF-I incorporated into biocompatible hydrogels based on OPF with gelatin microparticles. When delivered to osteochondral defects in rabbits, the best histological result was observed after 3 months* in vivo* with IGF-I-treated defects, while these benefits were not maintained when codelivered with TGF-*β*1 or when TGF-*β*1 was delivered alone, suggesting that in this* in vivo* model IGF-I was superior to TGF-*β*1 [40]. In the most recent work, the same authors found that while delivery of BMP-2 enhanced subchondral bone formation, dual delivery of IGF-I and BMP-2 in separate layers did not improve cartilage repair but they may synergistically enhance the degree of subchondral bone formation [42].

### 3.2. Nonviral Gene Delivery of Factors

Gene transfer via nonviral vectors (transfection) is based on the incorporation of DNA, either naked or complexed with cationic polymers or with cationic lipids (in polyplexes and lipoplexes), into the target population. Though considered as a safe method as it avoids the risk of acquiring replication competence and of insertional mutagenesis without inducing immune responses in the host, its use is limited by the low transfection efficiencies (40–50% maximum) and short-term transgene expression levels achieved [63–65]. While the use of hydrogels for controlled delivery of growth factors has been broadly exploited in the context of cartilage repair, their application as controlled delivery systems of gene transfer vectors has been mainly focused on the improvement of the efficiency of transfection and the durability of the expression of transgene in tissue engineering approaches in general, with a very limited number of studies reporting their use on cartilage repair [41]. An overview of the main materials used for the fabrication of hydrogels for controlled delivery of nonviral vectors is summarized in Table 2.

Local gene delivery via hydrogel scaffolds has been studied through the encapsulation of naked DNA during hydrogel formation [66, 67] using synthetic polymers such as PLGA [66] or pNIPAAm [67]. Although naked DNA achieved gene expression and guided repair* in vivo* [76], its low gene transfer efficiency and rapid diffusion of the DNA from the hydrogel network urged searching for alternative gene delivery systems like those based on the controlled release of DNA complexed in lipoplexes or polyplexes [74]. Gene delivery of polyplexes [41, 70–75] or lipoplexes [68, 69] has been studied by using different hydrogel systems including fibrin [69, 72], HA [70, 72–75], and PEG [41, 68, 70, 71] to target different cell populations including MSCs [71, 74, 75] in a variety of tissue engineering approaches (Table 2).

An important limitation of the incorporation of nonviral gene transfer vectors into hydrogels is the failure of loading high DNA concentrations because of their tendency to aggregate. Incorporation of nonviral vectors into hydrogel networks often results in their aggregation as a result of the soft, loose, and charged structures of polyplexes and lipoplexes [70, 73]. To solve such issues, a caged nanoparticle encapsulation technology has been designed [70, 73, 74]. Incorporation of lyophilized powder of polyplexes suspended in a medium containing sucrose and agarose into HA hydrogels significantly prevented their aggregation compared with direct encapsulation of DNA/polyethylene imine (PEI) control polyplexes [70, 73, 74].

The use of hydrogels as controlled gene delivery systems for cartilage repair is a very valuable but still developing strategy. Osteochondral and chondral units are especially promising tissues for polymeric gene delivery approaches because of the limited blood flow to the region which could cause problems in DNA polymer complex delivery and the potential for the delivered genes to induce differentiation of infiltrated MSCs [41]. Needham et al. [41] recently described an innovative approach by delivering DNA polyplexes from OPF hydrogel scaffolds for osteochondral injury repair. An OPF layered scaffold mimicking the native osteochondral tissue organization was simultaneously loaded with DNA polyplexes encoding the runt-related transcription factor 2 (RUNX2) or sex determining region Y-box 5, 6, and 9 (SOX trio) to generate bone and cartilage tissues, respectively, in a rat osteochondral defect model. At 6 weeks after implantation, combination of RUNX2 and SOX trio DNA led to a significantly improved healing ability compared with empty hydrogels or each factor alone [41].

### 3.3. Viral Gene Delivery of Factors

Gene transfer using viral vectors (transduction) is based on the natural cellular entry pathways of viruses from which they are derived, resulting in higher gene transfer efficiencies compared with nonviral vectors (80–90%) [47, 65, 77, 78]. The most common viruses manipulated for gene transfer purposes include adenoviruses [79–81], herpes simplex virus (HSV) [82], retro- and lentiviruses [83–85], and adeno-associated virus (AAV) [86–89]. Although gene transfer via viral vectors is highly efficient, the existence of patient-associated factors and physiological barriers (existence of neutralizing antibodies against the viral capsid and inhibition of transduction in the presence of specific anticoagulants) may interfere with the effective delivery, processing, and expression of transgene inside the target cells [47, 90]. Also, intra-articular injection of viral vectors can result in the rapid dispersion of the particles from the joint space and diffusion to nontarget sites, leading to reduced gene transfer efficiencies in cells recruited in the lesions [29, 91].

Controlled release of viral vectors from hydrogels allowing for a release pattern via a diffusion process may help overcome these issues. A summary of the main biomaterials used to produce hydrogels for controlled release of viral vectors in different tissue engineering approaches is shown in Table 3. Most of the work reporting the use of hydrogels as controlled delivery systems of viral vectors aimed at overcoming the limitations associated with these types of vectors in different tissue engineering approaches, with only a few publications focusing on cartilage repair [22, 29, 37].

Adenoviral, lentiviral, and rAAV vectors have been encapsulated in fibrin hydrogels, taking advantage of the low immunogenicity and biodegradability of this compound and showing sustained release profiles of the vectors and expression of the transgenes of interest in different cell targets [29, 93–95]. Most interestingly, Lee et al. [29] reported that release of an rAAV carrying TGF-*β* from diluted fibrin glue hydrogels resulted in enhanced production of TGF-*β* and higher levels of cartilage-specific gene expression in human MSCs (hMSCs) compared with undiluted hydrogels. This fact was attributed to the more open network structure from diluted fibrin glue hydrogels compared with undiluted ones resulting in the most efficient release of rAAV TGF-*β* vectors [29].

Self-assembling peptides RAD16-I in a pure (RAD) form or combined with HA (RAD-HA) have been also employed to release rAAV vectors as a means of genetically modifying hMSCs [37]. Such systems were capable of efficiently encapsulating and releasing rAAV in a sustained, controlled manner to effectively transduce the cells (up to 80%) without deleterious effects on cell viability (up to 100%) or on their potential for chondrogenic differentiation of the cells over time (up to 21 days) [37].

PEO- and PPO-based “smart” or “intelligent” self-assembling, temperature-sensitive copolymers have been also utilized as efficient rAAV-mediated delivery systems due to their capacity to form polymeric micelles and to undergo sol-to-gel transition upon heating [96, 97]. Specifically, encapsulation of rAAV vectors in poloxamer PF68 and poloxamine T908 polymeric micelles allowed for effective, durable, and safe modification of hMSCs via rAAV to levels similar to or even higher than those noted upon direct vector application (up to 95% of gene transfer efficiency) [44]. Of further note, these copolymers were capable of restoring the transduction of hMSCs with rAAV in conditions of gene transfer inhibition like in the presence of heparin or of a specific antibody directed against the rAAV capsid, enabling effective therapeutic delivery of a chondrogenic* sox9* sequence leading to enhanced chondrocyte differentiation of the cells [44]. Furthermore, various hydrogel composite structures based on alginate (AlgPH155) and poloxamer PF127 were prepared by cross-linking at either high (50°C; AlgPH155+PF127 [H]) or room temperature (AlgPH155+PF127 [C]) to encapsulate and release rAAV vectors [22]. Strikingly, hydrogels based on AlgPH155 alone had the highest initial burst of rAAV release while those cross-linked as AlgPH155+PF127 [C] had the most sustained release pattern, all leading to high transduction efficiencies in hMSCs (~80%) over an extended period of evaluation (up to 21 days) [22].

## 4. Conclusions and Outlook

The use of hydrogels as controlled delivery systems of bioactive molecules is a valuable strategy to achieve appropriate levels of a therapeutic factor into target places, circumventing possible limitations associated with their direct administration (frequency and amounts of required doses, inflammatory and host immune responses, and possible diffusion to nontarget locations). Hydrogel delivery platforms can be modulated by tuning some specific parameters from the hydrogel networks as composition, cross-linking density pore size, and degradation kinetics. Hydrogel networks are promising systems for cartilage tissue engineering approaches as they exhibit many intrinsic features mimicking the ECM from cartilage showing in many studies biocompatibility with key cell populations involved in cartilage regenerative processes as chondrocytes and MSCs.

Although promising technological advances have been reported to produce hydrogels as delivery systems of cartilage reparative factors, large efforts are still necessary to obtain adapted hydrogel-based delivery systems that may lead to successful clinical translation in patients integrating conditions of efficacy, durability, and safety. In this sense, many relevant parameters from the hydrogel platforms such as encapsulation efficiency, interaction with and stability of the biomolecule cargo, tensile strength, resistance against dilution, gene transfer efficiency, and biocompatibility need to be accurately optimized. It will be also very important to keep in mind that a successful system* in vitro* might not generate similar/sufficient or adapted effects in a native environment* in vivo*. It will thus be fundamental to test the adaptability of the hydrogel systems in sites of tissue damage using clinically relevant, complex orthotopic animal models of cartilage defect as a means of enhancing the natural repair processes. Yet, despite remaining challenges, recent advances in the development of hydrogels as controlled release delivery systems of cartilage reparative factors are promising, new avenues of research that may clearly improve cartilage repair in patients in a close future.

## Figures and Tables

**Table 1:** Biomaterials used in hydrogels to deliver growth factors.

Hydrogels	Growth factors	Systems	Targets	Incorporation	Release profile	References
Alginate	VEGF, PDGF-BB, TGF-*β*1		MSCs	Affinity interaction	Sequential release	[19]
	TGF-*β*3, BMP-2	Peptide-modified alginate	MSCs		Dual release	[18]

Fibrin	IGF-I	Clot	Chondrocytes/cartilage defect (horse)	Encapsulation	n.a.	[25, 27]
	TGF-*β*1	Fresh and platelet-rich plasma fibrin	MSCs	Immobilization with heparin	n.a.	[28]
	TGF-*β*1		MSCs	Encapsulation	Decreased release with higher fibrinogen component	[26]
	TGF-*β*1		n.a.	Conjugation	Sustained release	[30]

Hyaluronan	TGF-*β*1	HA hydrogel with alginate microspheres	MSCs	Loading in microspheres	Reduced burst effect and sustained release for 6 days	[24]
	BMP-2	Heparin-decorated HA hydrogel particles	MSCs	Immobilization with heparin	Zero-order release kinetics	[23]

Chitosan	TGF-*β*1	Chitosan/collagen	MSCs	Conjugation	Sustained release with minimal burst effect	[60]

PEG	IGF-I, BMP-2	OPF	Osteochondral defect (rabbit)	Loading in gelatin microparticles	n.a.	[42]
	IGF-I, TGF-*β*1				High TGF-*β*1 and low IGF-I burst release followed by controlled release for 28 days	[40]
	TGF-*β*1	OPF	Bovine chondrocytes	Loading in gelatin microparticles	Controlled release for 28 days	[39]
	TGF-*β*1	PEG-genipin hydrogel with PLGA microspheres		Loading in PLGA microspheres	Sustained release for 21 days and minor burst with microspheres embedded in hydrogels	[33]
	IGF-I, TGF-*β*1	Photopolymerized hydrogel	Chondrocytes	Loading in PLGA microspheres	Controlled release for 2 weeks	[38]

Self-assembling peptides	TGF-*β*1	KLD_12_	MSCs	Tether or adsorption	50% released after 21 days	[35]
	TGF-*β*1	(RADA)_4_, (KLDL)_3_	MSCs	Tether or adsorption	Effective release by absorption	[36]

pNIPAAm	TGF-*β*3	pNIPAAm-coAAc	MSCs	Entrapment	Initial burst and zero-order release profile after 7 days	[43]

PVA	IGF-I	PLGA microparticles in PVA hydrogels	s.c. implantation (athymic mouse)		Controlled release for 6 weeks	[34]

PEG: polyethylene glycol; pNIPAAm: poly(N-isopropylacrylamide); PVA: poly(vinyl alcohol); VEGF: vascular endothelial growth factor; PDGF-BB: platelet-derived growth factor BB; TGF-*β*: transforming growth factor beta; BMP: bone morphogenetic protein; IGF-I: insulin-like growth factor I; HA: hyaluronic acid; OPF: oligo(poly(ethylene glycol) fumarate); PLGA: poly(lactic/glycolic acid); KLD_12_: self-assembling peptide with AcN-(KLDL)_3_-CNH_2_ sequence; RADA: self-assembling peptide with RAD motif; KLDL: self-assembling peptide with KLD sequence; MSCs: mesenchymal stem cells; n.a.: not applicable; s.c.: subcutaneous.

**Table 2:** Biomaterials used in hydrogels to deliver nonviral vectors.

Nonviral vectors	Materials	Systems	Genes	Release profiles	Efficacy	Targets	References
Naked DNA	PLGA	Injectable implant	*luc*	Sustained release for 2 months	Sustained expression, tolerance *in vivo*	CV1 cells, s.c. injection (mouse)	[66]
	pNIPAAm	pNIPAAm-co-AAc nanogel	*luc*	Not reported	Effective internalization	hMSCs	[67]
			GFP			s.c. injection (mouse)	

Lipoplexes	PEG	Hydrogel modified with affinity peptides	*luc*	~59%, 75%, and 80% in K8, K4, and RGD hydrogels for 6 days	5- to 15-fold increased transfection with K8 and K4 hydrogels	HT1080 cells	[68]
			GFP, *luc*				
	Fibrin	Microspheres in fibrin gel	eNOS	100% release by 24 h from fibrin gels, slower release with microspheres	Enhanced angiogenesis	Ear ulcer model (rabbit)	[69]

Polyplexes	PEG	OPF porous scaffold	SOX trio	Not reported	Combination of RUNX2 and SOX trio DNA improved healing relative to empty hydrogels	Osteochondral defect (rat)	[41]
			RUNX2				
			SEAP	Not free diffusion of polyplexes in hydrogel	Reduced polyplexes aggregation, effective gene transfer	NIH 3T3 cells, chorionic chick embryo	[70]
		Hydrogel with nanosized micelles	*luc*	Not reported	Higher transfection efficiency in the presence of micelles	hMSCs	[71]
	Fibrin		*lacZ*	<1% released at 3 days	Effective gene transfer	Chorionic chick embryo	[72]
			VEGF				
			SEAP	Not free diffusion of polyplexes in hydrogel	Reduced polyplexes aggregation, effective gene transfer	NIH 3T3 cells, chorionic chick embryo	[70]
	HA	Porous HA hydrogel	Gluc	Sustained release for 14 days in the presence of collagenase I treatment (<25% release)	Reduced aggregation of polyplexes	HEK293T cell	[73]
			SEAP				
		Fibrin hydrogel	*lacZ*	<1% released at 3 days	Effective gene transfer	Chorionic chick embryo	[72]
			VEGF				
		Microporous HA hydrogel	Gluc	Controlled release for 10 days	Sustained transgene expression for up to 10 days	mMSCs	[74]
		MMP-degradable HA hydrogel	Gluc	Stiffer hydrogels resulted in lower release rates in buffer, collagenase I, and hyaluronidase	Higher N/P ratios lead to higher gene transfer efficiency but also higher toxicity	mMSCs	[75]
			SEAP	Not free diffusion of polyplexes in hydrogel	Reduced polyplexes aggregation, effective gene transfer	NIH 3T3 cells, chorionic chick embryo	[70]

PLGA: poly(lactic/glycolic acid); pNIPAAm-co-AAc: poly(N-isopropylacrylamide-co-acrylic acid); PEG: polyethylene glycol; HA: hyaluronic acid; OPF: oligo(poly(ethylene glycol) fumarate); MMP: matrix metalloproteinase; *luc*: firefly luciferase; GFP: green fluorescent protein; eNOS: endothelial nitric oxide synthase; SOX: sex determining region Y-box; RUNX2: runt-related transcription factor 2; *lacZ*: *E. coliβ*-galactosidase; VEGF: vascular endothelial growth factor; Gluc: Gaussia luciferase; SEAP: secreted embryonic alkaline phosphatase; K8: GCGYGK8 peptide; K4: GCGK4 peptide; RGD: Arg-Gly-Asp; N/P: polyplex nitrogen-to-phosphate ratio; s.c.: subcutaneous; hMSCs: human mesenchymal stem cells; mMSCs: mouse mesenchymal stem cells.

**Table 3:** Biomaterials used in hydrogels to deliver viral vectors.

Viral vectors	Materials	Systems	Genes	Release profiles	Efficacy	Targets	References
Adenoviral	Collagen	IgG complexation in collagen gel	GFP	Controlled release	70% transduction at day 1, decreasing thereafter	Rat aortic smooth muscle cells	[92]
			GFP	Slow release	Bioactivity decreased	Fibroblasts	[93]
	Fibrin		GFP	Slow release	Half-maximal activity at 45 h	Fibroblasts, i.m. injection (mouse)	[93]
			BMP-7		Bone formation at 4 weeks		
			*lacZ*	Sustained release for 192 h	Enhanced bioactivity	Fibroblasts	[94]

Lentiviral	Fibrin	Hydrogel complexed with hydroxyapatite nanoparticles		Initial burst of release (40% at 4 h), controlled released for 6 days (75%)	Expression reduced in the presence of HA, decline between days 9 and 35	HEK293T cells, s.c. injection (mouse)	[95]

rAAV	Fibrin	FG	GFP	Biphasic higher release at low fibrin concentration (100% released at 2 weeks)	High efficiency at low FG concentration, decline after 8 days	hMSCs	[29]
			TGF-*β*1				
	RAD-16-I	Self-assembling peptide hydrogel pure or combined with HA	RFP	Faster release at high peptide concentration (0.4%), complete release at 6–10 days except for RAD spheres at 0.4% with 90% of release only after 21 days	80% transduction efficiency with spheres at 0.4%, time-course decline of expression	hMSCs	[37]
			*lacZ*				
	Alginate	Alginate/poloxamer composite systems cross-linked at room temperature (AlgPH155+PF127 [C]) or high temperature (AlgPH155+PF127 [H])	*lacZ*	(AlgPH155+PF127 [C]) led to the most controlled release profile	Higher transduction efficiency with AlgPH155+PF127 [H]	hMSCs	[22]

rAAV: recombinant adeno-associated viral vector; RAD-16-I: self-assembling peptide with (RADA)_4_ sequence; FG: fibrin glue; HA: hyaluronic acid; AlgPH: sodium alginate; PF127: poloxamer F127; GFP: green fluorescent protein; BMP: bone morphogenetic protein; *lacZ*: *E. coliβ*-galactosidase; TGF-*β*: transforming growth factor beta; RFP: red fluorescent protein; i.m.: intramuscular; hMSCs: human mesenchymal stem cells.
